# Plasma tau predicts cerebral vulnerability in aging

**DOI:** 10.18632/aging.104057

**Published:** 2020-11-04

**Authors:** Jose L. Cantero, Mercedes Atienza, Jaime Ramos-Cejudo, Silvia Fossati, Thomas Wisniewski, Ricardo S. Osorio

**Affiliations:** 1 Laboratory of Functional Neuroscience, Pablo de Olavide University, Seville, Spain; 2 CIBERNED, Network Center for Biomedical Research in Neurodegenerative Diseases, Madrid, Spain; 3 Division of Brain Aging, Department of Psychiatry, New York University School of Medicine, New York, NY 10016, USA; 4 Alzheimer's Center at Temple, Lewis Katz School of Medicine, Temple University, Philadelphia, PA 19140, USA; 5 Departments of Neurology, Pathology and Psychiatry, Center for Cognitive Neurology, New York University School of Medicine, New York, NY 10016, USA

**Keywords:** aging, plasma tau, cerebral vulnerability, cortical thickness, FDG-PET

## Abstract

Identifying cerebral vulnerability in late life may help prevent or slow the progression of aging-related chronic diseases. However, non-invasive biomarkers aimed at detecting subclinical cerebral changes in the elderly are lacking. Here, we have examined the potential of plasma total tau (t-tau) for identifying cerebral and cognitive deficits in normal elderly subjects. Patterns of cortical thickness and cortical glucose metabolism were used as outcomes of cerebral vulnerability. We found that increased plasma t-tau levels were associated with widespread reductions of cortical glucose uptake, thinning of the temporal lobe, and memory deficits. Importantly, tau-related reductions of glucose consumption in the orbitofrontal cortex emerged as a determining factor of the relationship between cortical thinning and memory loss. Together, these results support the view that plasma t-tau may serve to identify subclinical cerebral and cognitive deficits in normal aging, allowing detection of individuals at risk for developing aging-related neurodegenerative conditions.

## INTRODUCTION

Tau is a multifunctional microtubule-associated protein that results from alternative splicing of the *MAPT* gene [1] and is mainly expressed in the axons of neurons [2]. Under normal conditions, tau protein exists in soluble forms and its primary function is to regulate the assembly and maintenance of the structural stability of microtubules [3–4]. However, in Alzheimer's disease (AD) and other tauopathies, tau protein is accumulated resulting in post-translational modifications, the most important of which is hyperphosphorylation [5], that ultimately lead to aggregation into neurofibrillary tangles and neuronal death [6].

Despite intense investigation, pathways leading to tau-mediated neurotoxicity remain largely unknown. Accumulated evidence suggests that soluble, rather than aggregated, are the most deleterious forms of tau. Accordingly, previous studies have supported a role of soluble tau species in cell dysfunctions, synaptic loss and neuronal death [7]. More specifically, soluble tau oligomers have been shown to induce synaptic and mitochondrial dysfunctions [8–10], playing a critical role in the initiation and propagation of tau pathology in a prion-like manner [11].

There is a broad consensus that total tau (t-tau) is a marker of axonal and neuronal damage [12, 13] that increases with age [14]. Although t-tau is not specific to AD [12], increased t-tau levels in cerebrospinal fluid (CSF) are thought to reflect the intensity of neurodegeneration in the prodromal and clinical stages of AD [15–17], and may help to differentiate individuals at risk for developing AD from controls [18]. In addition, CSF t-tau has been related to cerebral hypometabolism in AD [19], as measured by 2-[18F]fluoro-2-deoxy-D-glucose-positron emission tomography (FDG-PET), a surrogate marker of reduced synaptic activity [20, 21]. The association between increased t-tau and cerebral hypometabolism become stronger when abnormal levels of amyloid-beta 42 (Aβ_1-42_) coexist in AD patients [22–24] and cognitively normal older adults [25, 26].

Tau levels can be examined in living individuals by analyzing CSF samples or by administering tau-specific ligands for use with positron emission tomography (PET) [27, 28]. However, the high cost, insufficient accessibility, or invasiveness of these techniques limits their use for screening purposes in the general population. Accumulated evidence suggests that t-tau can also be reliably measured in blood plasma. While previous studies have shown elevated plasma t-tau concentrations in AD patients compared to controls [29–33], the lack or weak correlations between CSF and plasma tau levels [29, 34], and the considerable overlap between groups [29, 35] hinders clinical translation. One possible explanation for this lack of diagnostic sensitivity is that impaired blood-brain barrier (BBB) not only occurs in AD patients but also in normal elderly individuals with early cognitive dysfunctions [36], thereby affecting plasma t-tau concentration in an unpredictable manner.

Although plasma t-tau may not be valid for diagnosis of prodromal and clinical AD, its elevated concentration has been associated with patterns of cortical thinning across MCI patients [35], lower gray matter density across the aging-AD continuum [30], delayed recall in AD [33], and cognitive decline in MCI patients when combined with plasma Aβ_1-42_ [37]. However, it remains unknown whether increased plasma t-tau also parallels subclinical cerebral and cognitive deficits in normal aging. Interestingly, recent evidence has shown that plasma t-tau reflects brain soluble, extracellular tau levels in the absence of tau pathology [38], suggesting that increased plasma t-tau in normal aging may indirectly signal subtle neuronal damage before neurodegeneration occurs.

The current study has been specifically designed to gain insight into these questions. We first examined whether plasma t-tau levels were associated with structural and metabolic cortical changes in cognitively normal older adults, as revealed by variations in cortical thickness and cortical glucose metabolism respectively. Given the tight relationship between tau and cerebral glucose hypometabolism [19], we hypothesized that increased plasma t-tau would be associated with deficits in cortical glucose uptake rather than with patterns of cortical thinning, likely reflecting decreased synaptic activity preceding cell shrinkage, reduced dendritic extent, and/or synaptic loss in vulnerable aging. We next investigated whether plasma t-tau and tau-related changes in cortical glucose metabolism and/or cortical thickness were linked to subclinical cognitive deficits in aging. Based on previous findings suggesting that higher plasma t-tau is associated with lower memory performance in MCI/AD [35], our prediction was that increased plasma t-tau and tau-related structural/metabolic cortical changes would be associated with poorer memory outcomes in cognitively normal older adults. As circulating Aβ may be a moderator of these relationships, the potential main and confounding effect of plasma Aβ was also evaluated.

## RESULTS

### Higher plasma tau correlates with poorer memory and lower Aβ_1-42_ in aging

**Figure 1:**
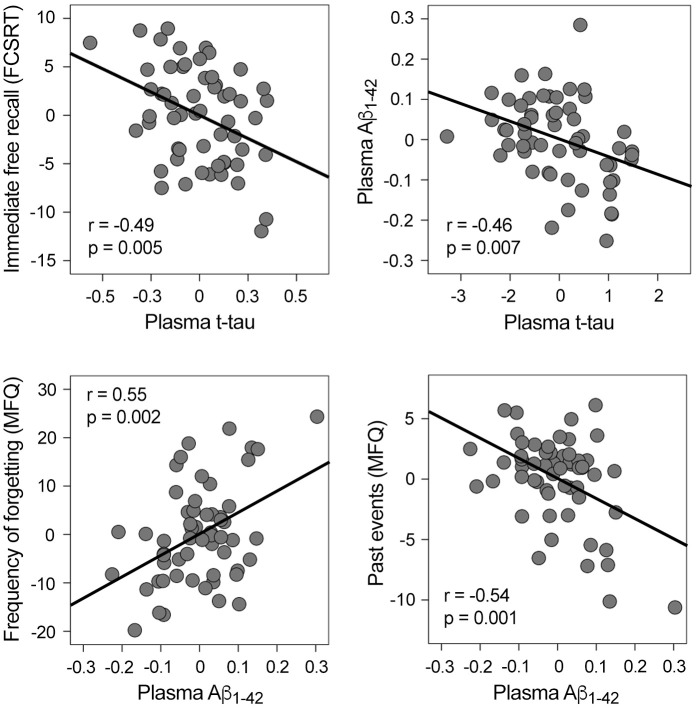
**Significant correlations between plasma t-tau, plasma Aβ_1-42_, and memory performance.** Variables included in the scatter plots correspond to the standardized residuals obtained from linear regression analyses. FCSRT: Free and Cued Selective Reminding Test; MFQ: Memory Functioning Questionnaire.

Plasma t-tau, plasma Aβ, and cognitive scores were normally distributed, thus allowing for the use of parametric statistical tests. Table 1 shows the characteristics of the cohort. While increased t-tau was associated with worse immediate free recall (r = -0.49, p = 0.005; β = -0.39, CI_95%_ = [-16.2 -3.0]), higher Aβ_1-42_ was related to more self-reported memory complaints (Figure 1). More specifically, increased Aβ_1-42_ was associated with higher frequency of forgetting (r = 0.55, p = 0.002; β = 0.44, CI_95%_ = [17.7 71.3]) and poorer memory of past events (r = -0.54, p = 0.001; β = -0.47, CI_95%_ = [-26.3 -7.2]). Plasma t-tau or Aβ species were unrelated to other cognitive domains.

**Table 1:** Characteristics of the study sample.

Age (yrs)	67.7 ± 3.4 (62-78)
Sex (M/F)	27/30
CDR	0
MMSE	29 ± 1.4 (26-30)
MFQ-forgetting	40 ± 11 (20-63)
MFQ-past events	17.7 ± 3.9 (8-24)
FCSRT-immediate free recall	24.7 ± 5.3 (12-36)
TOL	399.2 ± 145.5 (186-814)
PSI	116.8 ± 11 (89-142)
Total tau (pg/ml)	3.1 ± 1.5 (0.8-6.9)
Aβ_1-40_ (pg/ml)	231.2 ± 35.6 (165.7-331.6)
Aβ_1-42_ (pg/ml)	25.5 ± 6.5 (14-46.7)

M: male, F: female. CDR: Clinical Dementia Rating; MMSE: Mini Mental State Examination; MFQ: Memory Functioning Questionnaire; FCSRT: Free and Cued Selective Reminding Test; TOL: Tower of London; PSI: processing speed index (PSI) from the Wechsler Adult Intelligence Scale-III. Results are expressed as mean ± standard deviation (min-max).

Based on evidence supporting the synergy between cerebral Aβ and tau in both AD [46, 47] and normal aging [48], we examined if this relationship extended to blood plasma in cognitively normal elderly subjects. Our analyses showed that t-tau and Aβ_1-42_ significantly predicted each other (*t-tau*: r = -0.46, p = 0.007; β = -0.35, CI_95%_ = [-0.3 -0.05]; *Aβ_1-42_*: r = -0.4, p = 0.007; β = -0.37, CI_95%_ = [-1.3 -0.2]) (Figure 1). In contrast, t-tau and Aβ_1-40_ did not reveal a significant association.

### Higher plasma tau correlates with widespread reduction of cortical glucose uptake and thinning of the temporal cortex in aging

**Figure 2:**
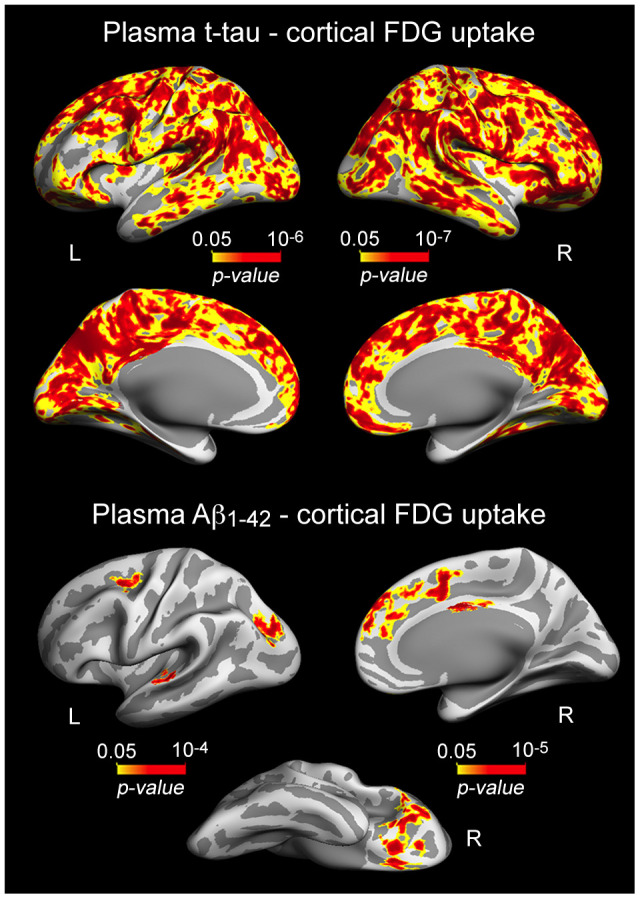
**Significant associations between increased plasma t-tau/Aβ_1-42_ and lower cortical FDG uptake.** Results are represented on inflated cortical surfaces. Left (L) and right (R). The color scale bar illustrates the range of significant p-values.

Having established the relationship between plasma t-tau and Aβ_1-42_ or memory performance, we next investigated whether t-tau or Aβ levels were related to variations in cortical thickness and/or cortical FDG uptake. We showed robust associations between either increased plasma t-tau or Aβ_1-42_ and lower glucose uptake, although they both differed in cortical extension and regions affected (Figure 2, Table 2). T-tau-FDG associations spread over the entire neocortex, showing strongest correlations over posterior cingulate (left: p = 10^-6^; right: p = 10^-7^), while Aβ_1-42_-FDG correlations were limited to superior parietal (left: p = 10^-4^), superior frontal (left: p = 10^-3^; right: p = 10^-5^), superior temporal (left: p = 10^-4^), lateral orbitofrontal (right: p = 10^-4^), and posterior cingulate (right: p = 10^-5^). We did not find significant associations between plasma Aβ_1-40_ and cortical FDG or between either increased t-tau or Aβ_1-42_ and higher cortical FDG uptake.

**Table 2:** Correlations between increased plasma levels of t-tau/Aβ_1-42_ and decreased cortical FDG uptake.

**Cortical region**	**CS (mm^2^)**	**r**	**p**
t-tau			
L posterior cingulate	54592	0.62	10^-6^
L superior temporal	119	0.32	10^-2^
R posterior cingulate	60700	0.61	10^-7^
R inferior temporal	175	0.35	10^-3^
Aβ_1-42_			
L superior parietal	756	0.49	10^-4^
L superior frontal	304	0.46	10^-3^
L superior temporal	232	0.51	10^-4^
R lateral orbitofrontal	1896	0.56	10^-4^
R superior frontal	1612	0.57	10^-5^
R posterior cingulate	205	0.66	10^-5^

CS: cluster size; it refers to the extent of significant correlations between plasma t-tau/Aβ_1-42_ and cortical FDG uptake. Left (L) and right (R) cortical hemisphere. r: Pearson correlation coefficient; p: exact p-value (corrected for multiple comparisons).

**Figure 3:**
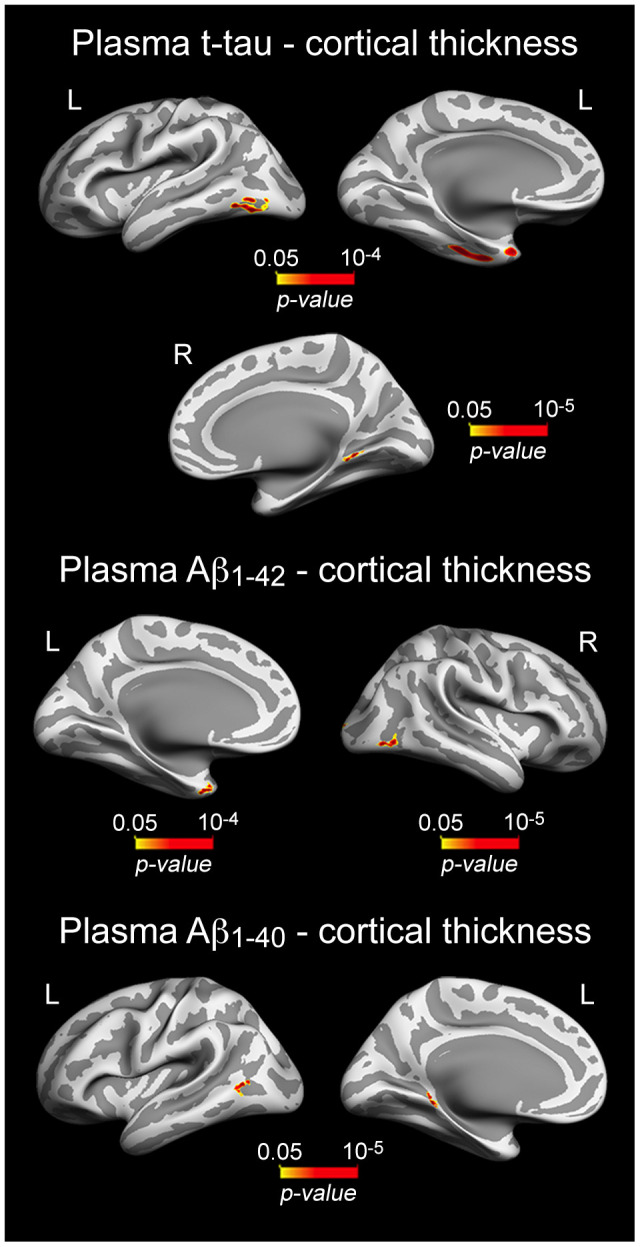
**Significant associations between increased plasma t-tau/Aβ_1-40_/Aβ_1-42_ and patterns of cortical thinning.** Results are represented on inflated cortical surfaces. Left (L) and right (R). The color scale bar illustrates the range of significant p-values.

As neuronal loss is relatively limited in normal aging [49, 50], it is reasonable to assume that correlations between plasma t-tau and cortical thickness should affect cortical regions particularly vulnerable to aging rather than widespread cortical networks. Accordingly, we showed that increased t-tau was associated with thinning of local aspects of the temporal lobe bilaterally (10^-5^ < p < 10^-4^) (Figure 3, Table 3). Significant correlations between plasma Aβ and cortical thickness were Aβ peptide-dependent. While increased Aβ_1-40_ was significantly associated with thinning of different aspects of the left temporal lobe (10^-5^ < p < 10^-4^), higher Aβ_1-42_ was related to thinning of the left temporal pole (p = 10^-4^) and right lateral occipital cortex (p = 10^-5^) (Figure 3, Table 3).

**Table 3:** Correlations between increased plasma levels of t-tau/Aβ species and patterns of cortical thinning.

**Cortical region**	**CS (mm^2^)**	**r**	**p**
t-tau			
L inferior temporal	410	0.52	10^-4^
L fusiform gyrus	336	0.45	10^-4^
L temporal pole	123	0.36	10^-4^
R lingual gyrus	85	0.49	10^-5^
Aβ_1-40_			
L middle temporal	133	0.56	10^-5^
L parahippocampal	59	0.47	10^-4^
Aβ_1-42_			
L temporal pole	115	0.5	10^-4^
L parahippocampal	59	0.47	10^-4^
R lateral occipital	176	0.6	10^-5^

CS: cluster size; it refers to the extent of significant correlations between plasma markers and cortical FDG uptake. Left (L) and right (R) cortical hemisphere. r: Pearson correlation coefficient; p: exact p-value (corrected for multiple comparisons).

### Relationship between plasma t-tau, cortical glucose metabolism, cortical thickness and memory in aging

**Figure 4:**
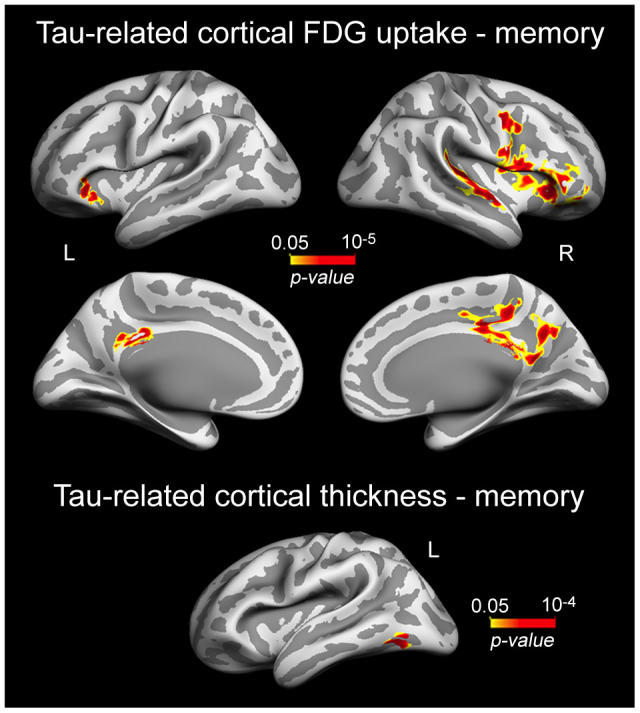
**Significant associations between plasma t-tau-related reductions of cortical FDG uptake/cortical thinning and memory performance.** Results are represented on inflated cortical surfaces. Left (L) and right (R). The color scale bar illustrates the range of significant p-values.

Based on previous evidence linking tau-associated neural dysfunctions to brain atrophy and worse memory in normal aging [48], we hypothesized that patterns of cortical thinning/decreased cortical FDG uptake related to plasma tau/Aβ species would also be associated with lower memory performance in cognitively normal older individuals. Results showed that correlations were limited to the left inferior temporal gyrus (p = 10^-4^) for cortical thinning, and spread over the right superior temporal (p = 10^-5^), and bilaterally over lateral orbitofrontal (left, p = 10^-5^; right, p = 10^-5^) and cingulate cortices (left, p = 10^-3^; right, p = 10^-4^) for cortical hypometabolism (Figure 4, Table 4).

**Table 4:** Correlations between plasma t-tau related cortical changes and memory deficits.

**Cortical region**	**CS (mm^2^)**	**r**	**p**
t-tau (cortical thinning)			
L inferior temporal	297	0.46	10^-4^
t-tau (cortical hypometabolism)			
L lateral orbitofrontal	460	0.52	10^-5^
L posterior cingulate	277	0.43	10^-3^
R lateral orbitofrontal	3971	0.49	10^-5^
R posterior cingulate	15731	0.48	10^-4^
R superior temporal	1076	0.47	10^-5^

CS: cluster size; it refers to the extent of significant correlations between t-tau related cortical changes and immediate free recall (FCSRT). Left (L) and right (R) cortical hemisphere. r: Pearson correlation coefficient; p: exact p-value (corrected for multiple comparisons).

**Figure 5:**
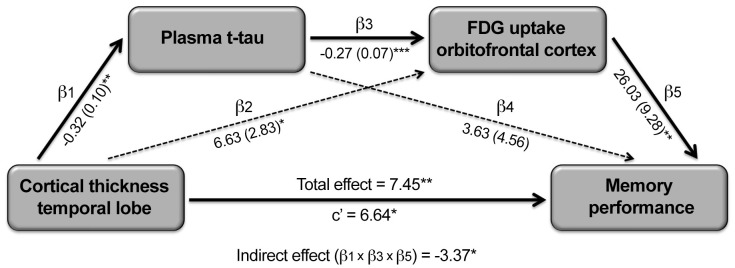
**Serial mediating role of plasma t-tau and cortical FDG uptake on the relationship between cortical thinning and memory deficits in aging.** Path analysis showing the serial mediation of higher plasma t-tau and lower FDG uptake in the orbitofronal cortex on the relationship between cortical thinning in the temporal lobe and memory deficits. Numbers along paths are unstandardized regression coefficients with the standard deviation in parenthesis. Asterisks indicate that the direct path as well as the total and indirect effect were statistically significant (*p < 0.05; **p < 0.005; ***p < 0.001). Thicker lines refer to the paths intervening in the three-way indirect effect.

As synaptic dysfunctions caused by tau oligomers are thought to precede neuronal loss in AD models [8], and decreased cerebral glucose metabolism relates to reduced synaptic activity [20, 21], we hypothesized that the association between t-tau-related cortical thinning and memory loss would be mediated, at least partially, by t-tau, cortical FDG uptake and/or by the serial relationship between t-tau and cortical FDG uptake. To formally test this hypothesis, we performed single and serial mediation analyses. None of the single-mediator analyses showed significant indirect effects. On the contrary, the serial mediation model revealed a significant four-path indirect effect when FDG consumption in the left orbitofrontal cortex was introduced as second mediator (Figure 5). This model showed a significant total effect of inferior temporal atrophy on memory (β = 7.45, CI_95%_ = [1.84 13.05]). As expected, inferior temporal atrophy was associated with increased t-tau (β = -0.32, CI_95%_ = [-0.54 -0.10]), t-tau was negatively correlated with FDG uptake in the left orbitofrontal cortex after adjusting by inferior temporal thickness (β = -0.27, CI_95%_ = [-0.40 -0.13]), and decreased left orbitofrontal FDG uptake was associated with worse memory after controlling for the effects of inferior temporal thickness and t-tau (β = 26.03, CI_95%_ = [7.21 44.85]). According to our hypothesis, the four-path indirect effect was statistically significant (β = 2.21, CI_95%_ = [0.45 6.35]), differed from the indirect effect due to t-tau (β = -3.37, CI_95%_ = [-10.43 -0.21]), and showed a significant size effect (P_M_ [CI_95%_] = 0.30 [0.05 1.70]). However, the mediation was not complete because the direct effect, although lower than the total effect, remained significant (β = 6.64, CI_95%_ = [0.89 12.39]), suggesting that other mediators could partially explain the association between inferior temporal atrophy and worse memory in normal aging. The serial mediation effect was limited to decreased FDG uptake in the left orbitofrontal cortex. Alternative models modifying the order of the independent variable and mediators did not reveal significant indirect effects.

## DISCUSSION

This study sought to determine the potential of plasma t-tau for detecting cerebral vulnerability in aging. Results revealed that increased concentration of plasma t-tau was associated with widespread reduction of cortical glucose uptake, thinning of the temporal lobe, and memory deficits. In particular, the generalized tau-related decrease of cortical glucose consumption emerged as a determining factor of the relationship between cortical thinning and memory loss. Moreover, plasma t-tau-related deficits in brain and cognition markedly differed from those associated with plasma Aβ. Overall, these results support the view that plasma t-tau is able to track subclinical cerebral and memory deficits in normal aging, which may serve for detecting incipient neuronal damage in primary care settings, facilitating the enrollment of high-risk individuals into dementia prevention trials.

### Higher plasma t-tau is associated with memory deficits in aging

Evidence linking AD biomarkers to cognitive function in normal aging has mostly focused on Aβ pathology. Despite considerable heterogeneity of results, the association between amyloid burden and poor cognition is supported by both cross-sectional [51–53] and longitudinal studies [51, 54, 55]. Studies focused on the relationship between tau and cognition in normal aging have revealed a close link between tau pathology and lower episodic memory [56–59] and pattern-separation abilities [48], both functions governed by structures of the medial temporal lobe. These findings are consistent with evidence showing a relationship between tau deposition in the temporal lobe and hippocampal disconnection, which in turn is associated with worse episodic memory performance in normal aging [60]. The question arises as to whether plasma Aβ or tau measurements are also able to explain subclinical cognitive deficits in nondemented elderly subjects.

To our knowledge, this study represents the first evidence of association between plasma t-tau and cognitive aging. Previous research has revealed that higher plasma t-tau was correlated with worse memory performance in MCI/AD patients in cross-sectional [33, 35] and longitudinal studies [29, 61, 62]. Although the controversy still exists over whether neurofibrillary tangles or Aβ plaques are the first event in AD, evidence suggests that CSF Aβ_1-42_ may be the first biomarker to change in the progression to AD followed by tau pathology [63, 64], supporting associations between increased plasma Aβ_1-42_ and worse subjective memory that precede tau-related objective memory changes in normal aging. Accordingly, the late-life accumulation of Aβ and tau in plasma may serve to monitor cognitive decline in normal aging, paving the way to clinical trials focused on evaluating the efficacy of anti-Aβ and tau interventions to prevent cognitive decline in preclinical AD.

We further showed that increased t-tau was associated with worse memory performance whereas higher Aβ_1-42_ was related to more self-reported memory complaints, suggesting that plasma concentration of both AD-related proteins uncover different aspects of memory deterioration in aging. Accumulated evidence suggests that subjective memory complaints, in the absence of cognitive impairment, increase the dementia risk in the general population [65–68] and are related to cerebral deficits [69–72]. Importantly, recent findings have shown that Aβ_1-42_ were associated with t-tau levels, suggesting that elevated Aβ_1-42_ combined with subjective memory complaints may signal an increased risk for developing AD [73]. Previous studies have shown that elevated plasma Aβ_1-42_ at baseline and decreasing levels over time predicts conversion to AD [74, 75], supporting the hypothesis that plasma Aβ_1-42_ fluctuations might signal earlier neurologic changes that may eventually culminate in dementia. In line with this hypothesis, our findings suggest that increased plasma Aβ_1-42_ may reflect higher cerebral vulnerability in aging, as revealed by its association with self-reported memory complaints, regional cortical thinning and reduced cortical glucose uptake.

Evidence suggests that Aβ_1-40_ has preference for cardiovascular aging while Aβ_1-42_ is mainly involved in development of AD [76], presumably explaining the lack of association between plasma Aβ_1-40_ and t-tau reported in the present study. Accordingly, Aβ_1-40_ is selectively deposited in leptomeningeal blood vessels in contrast to Aβ_1-42_ that is mostly found in senile plaques [77]. Moreover, studies assessing the association of plasma Aβ_1-40_ with cognitive function have not yielded consistent results [78], suggesting that aging-related cognitive deficits are mainly reflected in plasma t-tau and Aβ_1-42_ rather than in Aβ_1-40_.

### Higher plasma t-tau is associated with cortical deficits in aging

The present study revealed that higher plasma t-tau was associated with a global reduction of cortical FDG uptake. The association with cortical hypometabolism was more noticeable for plasma t-tau than for Aβ_1-42_, suggesting that plasma t-tau is a better biomarker of aging-related synaptic reduction than Aβ_1-42_. Interestingly, we further found that tau-related FDG reduction over right prefrontal cortex and bilateral posterior cingulate was associated with subclinical memory loss. While there is evidence linking increased plasma t-tau to hypometabolism in MCI [79] and AD patients [29], associations between tau-related cortical hypometabolism and aging-related memory deficits have not been thoroughly investigated. Our observations are consistent with recent evidence showing that tau-PET binding was associated with lower FDG uptake over posterior cingulate that, in turn, correlated with aging-related memory decline [25]. Mechanistically, tau-related associations with hypometabolism of posterior cingulate may be driven by the vulnerability of this region to Aβ burden [80] and/or by the selective reduction of the mitochondrial enzyme cytochrome oxidase in this region [81], likely featuring the earliest sign of energy hypometabolism in AD.

Previous research has shown that higher plasma t-tau is associated with cortical atrophy in MCI/AD patients [30, 35]. Our study extends these findings by linking increased plasma t-tau to patterns of cortical thinning in normal aging. We showed that tau-related patterns of cortical thinning were restricted to small patches of the temporal lobe critically affected in predementia stages, as the inferior temporal cortex [82], fusiform gyrus [83], lingual gyrus [84], and the temporal pole [85]. Interestingly, most associations between plasma t-tau or Aβ species were restricted to temporal cortices, reinforcing the view that circulating blood levels of both AD proteins are able to track the structural integrity of temporal lobes in aging [70]. Contrary to our results, recent research has revealed no significant association between plasma t-tau and volume/thickness of different cortical regions in a cohort of cognitively normal adults [86]. Different methodological aspects may contribute to account for discrepancies between these studies. First, samples of both studies are markedly different. Chiu and collaborators [86] examined cognitively normal middle-aged and older adults (age range: 45-95 yrs.), whereas the present study was focused on late life (62-78 yrs.). Second, our MRI analysis protocol included manual correction of misclassified cerebral tissues, which probably enhanced the reliability of cortical thickness measurements. Third, cortical thickness maps were smoothed using non-linear spherical wavelet-based de-noising schemes, which have previously shown greater specificity and sensitivity than Gaussian spatial filters for detecting local changes in cortical thickness [43]. Fourth, both studies employed different approaches to analyze cortical thickness. While we applied surface-based vertex-wise analysis, Chiu et al. [86] used ROI analysis in their study. Finally, ultra-sensitivity assays used for determining plasma t-tau also differed between the two studies. We used single molecule array (SIMOA)-based assays that rely on two epitopes and two antibodies (one for binding and other for tau detection), whereas Chiu et al.'s study [86] employed immunomagnetic reduction-superconducting quantum interference technology based on one antibody against one epitope for tau detection.

Our findings of association between increased plasma t-tau and metabolic/structural cortical changes support the idea that tau protein leaks from brain to bloodstream, revealing key aspects of the neuronal environment. The integrity of the dural lymphatic system, likely assisted by the water channel aquaporin 4 [87], seems to play a key role in clearance of extracellular tau and Aβ [88], as well as the importance of the BBB and endothelial function in the movement of tau from brain to blood [89], and possible effects of cardiovascular risk factors, trauma, or cerebrovascular pathology in AD development [90]. Emerging evidence supports a bidirectional relationship between brain extracellular tau and plasma tau. In this vein, administration of an anti-tau antibody to tau transgenic mice and patients with progressive supranuclear palsy, a tau-related neurodegenerative disorder, resulted in a dose-dependent increase in plasma tau that was bound to antibody, and correlated with the concentration of extracellular and soluble tau in the brain [38, 91]. Previous studies have also shown that lowering plasma tau levels via peritoneal dialysis reduces interstitial fluid tau levels in the brain [92], suggesting that enhancement of plasma tau clearance may be a potential therapeutic strategy for tauopathies. A better understanding of reciprocal influences between brain extracellular tau and plasma tau appears to be critical to monitor the efficacy of anti-tau antibody therapy in elderly subjects at risk for developing AD.

### The association between plasma t-tau and cortical hypometabolism mediates the relationship between cortical thinning and memory deficits in aging

Associations between cerebral tau load and memory are well documented in both normal aging [57] and AD [93]. However, whether this relationship is biologically plausible with plasma t-tau, and whether it is indirectly mediated by other factors in late life are unexplored questions. Our study revealed that causal relationships between cortical atrophy and memory deficits in aging are more complex than previously thought. We showed that increased plasma t-tau and lower glucose uptake in the orbitofrontal cortex was a determining factor of the association between t-tau-related cortical atrophy and memory deficits, suggesting that plasma t-tau and metabolism of the orbitofrontal cortex may play a critical role in monitoring aging-related memory decline. These results are supported by previous evidence showing that higher CSF tau concentrations predict lower cerebral glucose metabolism, and that FDG hypometabolism acts as a mediator between CSF tau and cognitive impairment in MCI/AD [94]. Our results further highlighted that the causal sequence of events leading to cognitive deficits may differ from normal aging to AD. Whereas cortical atrophy has been suggested to act as a mediator in the relationship between tau and cognition in AD patients [95], we showed that the association between plasma t-tau and cortical hypometabolism determined the relationship between cortical loss and memory deficits in normal aging. Further research is needed to elucidate the complementary role of different tau measurements on cognitive decline in aging and different AD stages, and to determine to what extent this role may eventually change with disease.

Compared to other studies [29–33, 35, 37, 61, 62, 79, 86], we have provided converging evidence from structural MRI, FDG-PET, cognitive performance, and mediation analysis, supporting that plasma t-tau is able to identify subclinical cerebral and cognitive deficits in normal aging. To our knowledge, this is also the first study to compare aging-related associations between structural/metabolic brain changes and plasma t-tau and Aβ species. In summary, plasma t-tau levels were associated with widespread reductions of cortical glucose uptake, patterns of cortical thinning affecting the temporal lobe, and subclinical memory deficits in cognitively normal elderly subjects. Importantly, plasma t-tau-related deficits in brain and cognition markedly differed from those associated with plasma Aβ. Moreover, tau-related reductions of glucose consumption in the orbitofrontal cortex appeared as a determining factor of the relationship between cortical thinning and memory loss.

## MATERIALS AND METHODS

### Participants

Fifty-seven cognitively normal elderly subjects, recruited from senior citizen’s associations, health-screening programs, and hospital outpatient services, were enrolled in the study. All participants were volunteers from different aging/AD research programs conducted in the Laboratory of Functional Neuroscience at Pablo de Olavide University (Seville, Spain). They showed normal cognitive performance in the neuropsychological tests relative to appropriate reference values for age and education level, and no medical illnesses or medications that affected cognition. All individuals showed a global score of 0 (no dementia) in the Clinical Dementia Rating (CDR), normal global cognitive status in the Mini Mental State Examination (MMSE) (scores ≥ 26), and normal independent function –assessed by the Spanish version of the Interview for Deterioration in Daily Living Activities [39]. Depression was excluded (scores ≤ 10) by the Geriatric Depression Scale [40]. All participants gave informed consent to the experimental protocol approved by the Ethical Committee for Human Research at the University Pablo de Olavide according to the principles outlined in the Declaration of Helsinki.

### Neuropsychological assessment

A neuropsychological battery covering memory, executive functioning, and processing speed was administered to all participants. Subjective memory complaints were evaluated with the Memory Functioning Questionnaire (MFQ) [41], while objective memory was assessed with the Free and Cued Selective Reminding Test (FCSRT). The Tower of London (TOL) and the processing speed index (PSI) from the Wechsler Adult Intelligence Scale-III (WAIS-III) were administered to evaluate executive function and processing speed, respectively.

### Plasma total tau and Aβ

Fasting blood samples were taken at 9:00-10:00 in all participants to control for potential circadian effects. Briefly, venous blood samples were collected in 10 ml K2-ethylenediaminetetraacetic acid (EDTA) coated tubes (BD Diagnostics), and immediately centrifuged (1989 g) at 4ºC for 5 min. Supernatant plasma was aliquoted into polypropylene tubes containing 250 μl of plasma mixed with 8.32 μl of a protease inhibitor cocktail (cOmplete Ultra Tablets mini, Roche), and stored at -80ºC until analysis. Plasma samples used in the present study were not previously thawed.

Plasma t-tau was measured with the SIMOA Tau 2.0 kit on the Simoa HD-1 analyzer (Quanterix Corporation, MA), following the manufacturer’s instructions. This method is based on digital array technology that measures t-tau in plasma or serum with a detection limit of 0.019 pg/ml. The assay utilizes a capture mouse monoclonal antibody that binds to an epitope in the mid-domain of tau and a detection mouse monoclonal antibody that binds to an epitope in the N-terminal region of the protein. This combination of antibodies reacts with both normal and phosphorylated tau, presumably measuring all forms of tau. All assays were run at 21ºC room temperature, in duplicate, and the average of the two measurements (pg/ml) was used for statistical analyses. Samples showing coefficients of variation higher than 20% were re-analyzed or excluded.

Plasma Aβ level was determined with a double-antibody sandwich ELISA (human Aβ_1-40_ and high sensitive Aβ_1-42_, Wako Chemicals, Tokyo, Japan). Samples and standards were incubated overnight at 8ºC with antibodies specific for Aβ_1-40_ or Aβ_1-42_ peptides, and the wells were read for absorption at 450 nm on a Victor 3 system (PerkinElmer, Waltham, MA), following the manufacturer’s instructions. Plasma Aβ level was measured in duplicate (50 μl), and the average of the two measurements (pg/ml) was used for statistical analysis. Both inter-assay and intra-assay coefficients of variation were kept below 10%. The detection limit for these assays was 1.04 pg/ml for Aβ_1-40_ and 0.54 pg/ml for Aβ_1-42_.

### MRI and FDG-PET acquisition

Structural brain images were acquired on a Philips Achieva 3T MRI scanner equipped with an 8-channel phased-array head coil (Philips, Best, Netherlands). A whole-brain T1-weighted magnetization prepared rapid gradient echo (MPRAGE) was acquired with the following parameters: sagittal slice orientation, repetition time (TR) = 2300 ms, echo time (TE) = 4.5 ms, matrix = 320 × 320, flip angle = 8°, voxel resolution = 0.8 mm^3^ isotropic, no gap between slices, acquisition time = 9.1 min. Head motion was minimized by using a head restraint system and placing foam padding around the subject's head. Participants were provided with headphones and foam earplugs to attenuate scanner noise.

FDG-PET brain images were acquired on a whole-body PET-CT Siemens Biograph 16 HiREZ scanner (Siemens Medical Systems, Germany) in 3D mode. Subjects fasted for at least 8 h before PET examination, and they were scanned at the same time of the day (8:00-9:00 am). Participants were injected with 370 MBq of FDG in a quiet, dimly-lit room. FDG-PET images were acquired in static mode 30 min after injection with scan duration of 10 min. FDG brain scans were corrected for attenuation, scatter and decay, smoothed, and reconstructed with 2.6 x 2.6 x 2 mm voxel resolution using back-projection filters.

### Estimation of surface-based cortical thickness and cortical glucose uptake

MRI data were processed using the analysis pipeline of Freesurfer v6.0 (https://surfer.nmr.mgh.harvard.edu/) that involves intensity normalization, registration to Talairach, skull stripping, white matter (WM) segmentation, tessellation of WM boundaries, and automatic correction of topological defects [42]. Pial surface misplacements and erroneous WM segmentation were manually corrected on a slice-by-slice basis to enhance the reliability of cortical thickness measurements. Individual cortical thickness maps were smoothed using non-linear spherical wavelet-based de-noising schemes, which have previously shown greater specificity and sensitivity for detecting local and global changes in cortical thickness [43].

Partial volume correction (PVC) of FDG-PET brain images was performed with the PMOD software v3.208 (PMOD Technologies Ltd., Switzerland) using the Müller-Gartner approach, and assuming a uniform 6 mm point spread function. To map PET scans onto individual cortical surfaces, FDG images were first co-registered to T1 scans. Next, PVC-cortical FDG images were transformed into standardized uptake value ratios (SUVr) using the gray matter of cerebellum (obtained with Freesurfer) as reference region. Next, resulting PVC-FDG cortical-to-cerebellum SUVr images were mapped into individual cortical surfaces with Freesurfer, and further smoothed with non-linear spherical wavelet-based de-noising schemes [43].

### Statistical analyses

We first assessed whether plasma t-tau, plasma Aβ species, and cognitive scores (FCSRT, TOL, PSI) deviated from normality using the Kolmogorov-Smirnov test with the Lilliefors correction. As the distribution of plasma t-tau was skewed, a log (base 10) transformation was applied prior to analysis. Next, regression analyses were conducted to evaluate whether plasma t-tau was associated with cognitive performance, adjusting by age, sex, Aβ_1-40_ and Aβ_1-42_. To avoid bias assessing the relationship between plasma t-tau and plasma Aβ (either Aβ_1-40_ or Aβ_1-42_), the alternative Aβ peptide was included as a confounding variable together with age and sex. These analyses were performed with R v3.0.1 (The R Foundation for Statistical Computing).

Vertex-wise regression analyses were further performed to examine associations between plasma t-tau and variations in cortical thickness/cortical FDG uptake. These analyses were also adjusted by age, sex, Aβ_1-40_ and Aβ_1-42_. Results were corrected for multiple comparisons using a previously validated hierarchical statistical model [44]. This procedure first controls the family-wise error rate in significant clusters by applying random field theory over smoothed statistical maps; and next controls the false discovery rate in vertices within significant clusters over unsmoothed statistical maps. A significant cluster was defined as a contiguous set of cortical surface vertices (≥ 90) that met the statistical threshold criteria (p<0.05 after correction for multiple comparisons). Further regression analyses were carried out using plasma Aβ as predictor (either Aβ_1-40_ or Aβ_1-42_), adjusted by age, sex, t-tau, and the alternative Aβ peptide. These analyses were performed with Freesurfer v6.0.

We next performed vertex-wise regression analysis to investigate whether tau-related changes in cortical thickness/cortical FDG uptake were associated with tau-related changes in cognition. To examine potential indirect relationships between plasma t-tau, cortical thickness, cortical glucose metabolism and cognition, we performed single and serial mediation analyses adjusted by age, sex, Aβ_1-40_ and Aβ_1-42_, using the “lavaan” package in R. Inference was determined by 95% bias-corrected bootstrap confidence intervals from 10,000 bootstrap samples, and the ratio of the indirect effect to the total effect (P_M_) was used as a measure of the effect size [45].
